# Three-Dimensional Expansion of a Dynamic Programming Method for Boundary Detection and Its Application to Sequential Magnetic Resonance Imaging (MRI)

**DOI:** 10.3390/s120505195

**Published:** 2012-04-26

**Authors:** Da-Chuan Cheng, Jui-Teng Lin

**Affiliations:** 1 Department of Biomedical Imaging and Radiological Science, China Medical University, Xueshi Road 91, Taichung 404, Taiwan; 2 New Vision Inc., 268-1 (11F), Han Sheng E Road, Banciao, New Taipei City 22066, Taiwan; E-Mail: jtlin55@gmail.com

**Keywords:** boundary detection, dynamic programming, MRI, femoral artery

## Abstract

This study proposes a fast 3D dynamic programming expansion to find a shortest surface in a 3D matrix. This algorithm can detect boundaries in an image sequence. Using phantom image studies with added uniform distributed noise from different SNRs, the unsigned error of this proposed method is investigated. Comparing the automated results to the gold standard, the best averaged relative unsigned error of the proposed method is 0.77% (SNR = 20 dB), and its corresponding parameter values are reported. We further apply this method to detect the boundary of the real superficial femoral artery (SFA) in MRI sequences without a contrast injection. The manual tracings on the SFA boundaries are performed by well-trained experts to be the gold standard. The comparisons between the manual tracings and automated results are made on 16 MRI sequences (800 total images). The average unsigned error rate is 2.4% (SD = 2.0%). The results demonstrate that the proposed method can perform qualitatively better than the 2D dynamic programming for vessel boundary detection on MRI sequences.

## 1. Introduction

Many image edge detection methods have been proposed in the last two decades [1,2]. They mainly differ in the types of smoothing filters that are applied and how the edge strength measures are computed. Edge detection methods allow broken or discontinuous edge lines. Boundary detections, however, are somehow different, as some applications do not allow discontinuous boundaries. Many types of methods can handle the vessel extraction problem. Several hundred papers are surveyed in [3], which consider vessel extraction in two-dimensional images. For instance, two-dimensional boundary detection utilizing graph-searching principles [4,5] has been studied frequently in medical image analysis. Boundary detections are sometimes transferred to an optimization problem that minimizes a given cost function [6–9]. Dynamic programming (DP) is an optimization tool often used in boundary detection [6,8,9]. Dynamic programming method performs well if the boundaries are visible. The motivation of this study is raised from the observation that the femoral artery in the cross-sectional view in MRI sequences without contrast injection is sometimes invisible. The artery is visible if the blood flow velocity is large enough that it appears in contrast compared to the surrounding tissue. This is most often an MRA image sequence. If the blood flow velocity is small during a short time period, the artery has limited contrast and is not visible. Under this extreme situation, the 2D DP fails to detect its boundary because it is hard to obtain a feature, normally the gray-level gradient, to represent the boundary. Our previous study has applied the local contrast to add additional information to handle extreme cases [10]. It still used local information but not information between two succeeding images. A relative study proposed a graph-based method to find an optimal surface in volumetric images [11]. This article is most related to our method, but it differs in principal. Good reviews are reported in other studies [12,13], in which methods are considered or models are represented in three dimensions. The methods treated the three-dimensional vessel geometry problems, in their issues vessel branching is a major problem to be solved. This is, however, not the major problem in our study. We do not consider three-dimensional vessel geometry. Instead, we focus on the vessel cross-sectional lumen changing with respect to a heart cycle at the same place. The lumen area must be quantified accurately. Moreover, the MRA images without a contrast medium sometimes present difficulty when visualizing vessel boundaries. Previous methods [3,12,13] are not suitable to solve our problem. This is for two reasons: (1) Our MRI data have vessel images, in which some of them the boundaries are vague and hard to recognize, even by human beings; (2) Our goal is to achieve a high accuracy on vessel's crossing area quantization. The goals of previous 3D methods are more suitable for visualization on vessels 3D structure, although the quantification is also possible if vessel boundaries are obtained.

To solve this problem, we propose a 3D-expansion of DP that can seek an optimal surface in a 3D matrix. This method can search succeeding boundaries in an image sequence, as succeeding boundaries form a surface in a 3D matrix. The relationship between two succeeding boundaries is thus considered in the smoothness constraint. To investigate the performance of this method, we design experiments to calculate its accuracy. The significance of this paper is two-fold: (1) we propose a 3D-expansion of DP which can locate an optimal surface among a 3D matrix; (2) we explore the best parameter set of the proposed 3D expansion of DP for round shape objects' boundary detection, and its unsigned relative error is reported.

The rest of this paper is organized as follows. In Sections 2 and 3, we introduce the 2D and 3D-expansion of DP, respectively. In Section 4, we address our experimental designs. In Section 5, we illustrate the experimental results. We then discuss the proposed method and give a conclusion in Sections 6 and 7.

## 2. Methods

### 2.1. 2D Dynamic Programming

In computer science, DP is a method for solving complex problems, which has similar properties that can be broken down into simpler sub-problems. Therefore, to solve a problem is then transformed to solve different parts of its sub-problems. However, many sub-problems are actually the same. The DP solves each sub-problem once and the result is saved for solving next similar sub-problem. Thus DP reduces the number of computations. A good tutorial on DP can be found in [14]. This approach was often used to find the contour of an object such as a road [15], and it has many applications on boundary detection in medical images [6,8–10,16–18]. A study has shown that DP outperforms some edge detectors against noise, including the Canny and Prewitt detector [19]. If the object to be detected contains noise, the DP might therefore be a good detection method, as for an MRI breast mass segmentation [20]. Similarly, DP is suitable for boundary detection in noisy images, including sonography [8,21] and MRI vessel segmentation without a contrast injection [10]. The 2D boundary detection using DP can be transformed to an optimization problem seeking an optimal path in a feature image. The searching direction is performed from one side to its opposite side. Converting a closed contour from its center in the Cartesian coordinate to the polar coordinate is thus required [22] for closed contour detection.

Let the feature image saved in a 2D matrix be *F, F* ∈ ℜ^*M*×*N*^. The boundary detection problem is then transformed to an optimization problem that searches for an optimal path. Assume the searching direction is from left to right. The optimization function can be defined to find the optimal path having the following global minimum:

(1) $$y_{x}^{*}=\arg\min\sum_{x=1}^{N}\left\{F(y_{x})|x=1,2,3,\cdots N\right\}$$

subject to some constraints, where *y_x_* is the y-coordinate on the *x*-th column in matrix F, and *y_x_* and *y*_*x*+1_ are neighborhood. This optimization function can thus be reformulated to implement DP with respect to an iterative cost function:

(2) $$C(x,y)=\min_{j\in (-d,d)}C(x-1,y+j)+F(x,y)+\alpha |j|$$

subject to 1 ≤ *x* ≤ *N*, 1 ≤ *y* ≤ *M*, where *α* is a weighting parameter controlling the smoothness of the searched path and *d* is the maximum distance between two connected nodes. *C*(*x,y*) is a two-dimensional cost map. The global optimization problem is the same as its subproblem *C*(*x* − 1,*y*), *C*(*x* − 2,*y*) and *vice versa*. We set *C*(1,*y*) = *F*(1,*y*), as a boundary condition. The optimal index j* can be determined by the following equation:

(3) $$j^{*}=\arg\min_{j\in (-d,d)}C(x-1,y+j)+\alpha |j|$$

The index can be stored in the 2D coordinate matrix *Y*(*x,y*) = *y* + j*, which is a pointer indicating a point on the previous column (*x* − 1). The cost map and path links are thus constructed column-wise from left to right on the feature matrix F. After construction, the optimal path can be found by tracing the path link backwards on the last column (*x* = *N*), which has the global minimum. There are some notable variations of the 2D DP, including the dual- [8] and multi-path DP [23].

### 2.2. 3D-Expansion of Dynamic Programming

**Figure 1.**
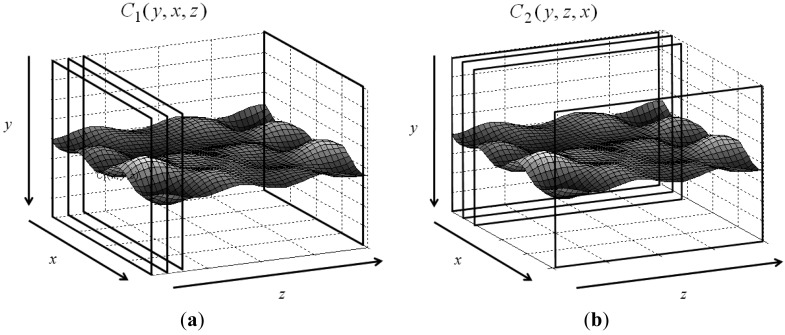
The 3D matrix R contains the feature of an image sequence. The structure of the accumulation matrices (**a**) C_1_ and (**b**) C_2_ is organized in different orientations.

Let the 3D matrix R have size *M* × *N* × *P*, where M and N are the numbers of rows and columns, and P denotes its depth. The volumetric matrix contains feature image sequences of interest. Values in R are normalized, *i.e.*, 0 ≤ *R*(*y,x,z*) ≤ 1, where y, x, and z are indices of the corresponding dimensions. Assume that features are saved in the 3D matrix and the goal is to find an optimal surface having the shortest path and lowest value summation from one side on the z-axis to another side with some given constraints. Figure 1 shows the 3D matrix R and the surface to be detected. The typical constraint in DP controls the smoothness or continuity of the sought surface. Assume the searching direction is from x = 1 to x = N and z = 1 to z = P. Two parameters control the smoothness: *d*_1_ ≥ |*y_x_* − *y*_*x*−1_| controls the smoothness on the *x* − *y* plane *N* × *M*, and *d*_2_ ≥ |*y_z_* − *y*_*z*−1_| controls the smoothness on the *x−z* plane *N* × *P*. The parameters allow the maximum distance between two connected nodes. The *y*-coordinate (*y*(*x,z*)) denotes the height of the optimal surface, which can be found via an optimization problem:

(4) $$y_{x,z}^{*}=\arg\min\sum_{x=1}^{N}\sum_{z=1}^{P}\left\{R(y_{x,z}\right)|1\leq x\leq N,1\leq z\leq P\}$$

subject to the constraints |*y_x_* − *y*_*x*−1_| ≤ *d*_1_ and |*y_z_* − *y*_*z*−1_| ≤ *d*_2_.

**Figure 2.**
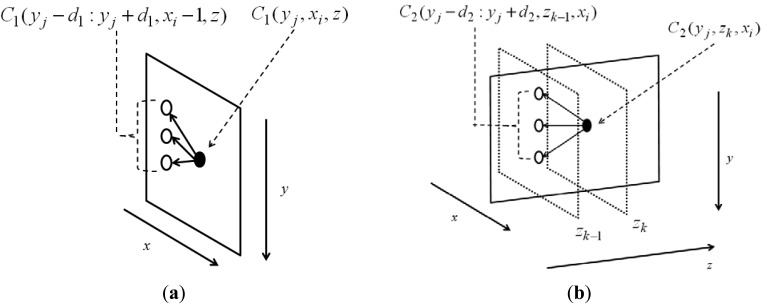
The accumulation matrices C_1_ and C_2_. The dimensions of (**a**) C_1_ and (**b**) C_2_ is same as those of R.

This optimization problem minimizes the iterative cost functions as such:

(5a) $$C_{1}(y,x,z)=\underset{-d_{1}\leq i\leq d_{1}}{\min}(C_{1}(y+i,x-1,z)+\alpha_{1}|i|)+R(y,x,z)$$

for 1 ≤ *x* ≤ *N* and 1 ≤ *z* ≤ *P*.

(5b) $$C_{2}(y,z,x)=\underset{-d_{2}\leq i\leq d_{2}}{\min}(C_{2}(y+i,z-1,x)+\alpha_{2}|i|)+C_{1}(y,x,z)$$

for 1 ≤ *z* ≤ *P* and 1 ≤ *x* ≤ *N*, where *C*1 and *C*_2_ are accumulation matrices of size *M* × *N* × *P* and *M* × *P* × *N*, respectively. Figure 2 shows their organizations. The initializations of *C*1 and *C*_2_ are thus:

(6a) $$C_{1}\left(*,1,z\right)=\text{R}\left(*,1,z\right)\;\text{for}\;1\leq z\leq P$$

(6b) $$C_{2}\left(*,1,x\right)=\text{R}\left(*,1,x\right)\;\text{for}\;1\leq x\leq N$$

During the accumulation process, the indices must be stored in Y_1_(*x,y,z*) for C_1_ and Y_2_ (*x,y,z*) for C_2_ for the backwards tracing [8]. After the minimization process, the accumulation matrix *C*_2_ contains the minimal value in the last column. In the backwards tracing, the y-coordinates of the optimal surface can be obtained sequentially. The minimal value in the last column is found using $y*(x,\text{end})=\underset{y}{\min}C_{2}(y,\text{end},x)$.

The remaining y- coordinates in the same index *x* can be traced backwards: y*(*x,end* − 1) = *Y*_2_(y*(*x,end*),*x,end*). The node in C_1_ searches for a node in its previous column (*x_i_* − 1), which makes the accumulation minimal and saves the optimal path link in another 3D matrix Y_1_, shown in Figure 2(a). During this process, the connection is considered only at the same depth z. The possible searching range is denoted by *d*_1_ and weighted by *α*_1_; both control the smoothness of the surface on *xy*-plane. Though the searching process is determined by the node value on its previous column plus the smoothness *α*_1_|*i*|, as in Equation (2a), the final value of the node is summed by the node value in the feature matrix *R*(*y,x,z*). Similarly, the node in C_2_ searches for the node in its previous column (*z_k_* − 1), which makes the accumulation minimal and saves the optimal path link in another 3D matrix Y_2_, shown in Figure 2(b). During this process, connection is considered only the same depth *x*. The possible searching range is denoted by *d*_2_ and weighted by *α*_2_; both control the smoothness of the surface on *yz*-plane. Though the searching process is determined by the node value on its previous column plus the smoothness *α*_2_|*i*|, as in Equation (2b), the final value of the node is summed by the node value in C_1_(*y,x,z*), in which the neighboring information on *xy*-plane has been already considered. The final C_2_ matrices thus contain the feature and neighboring information on both *xy*- and *yz*-planes.

Compared to the traditional DP, the 3D DP has the advantage that it connects curves in two planes (*xy*- and *yz*-planes). This property allows extracting the boundary even if the boundary is vague. Section 5 presents the examples. Notably, if the backwards tracing process is performed on the C_1_ matrix, it produces the same results as the traditional DP.

## 3. Experimental Design

### 3.1. Phantom Study

To investigate the accuracy of the proposed method, we design a phantom image sequence with added uniform distributed noise. The image gray level is normalized to be within the range [0,1]. The foreground (artery lumen) is set to 1, and the background is set to 0.4. The image has the size 41 × 41, with simulated artery lumens having radii ranging from 9 to 12 pixels (with step 1) and back to 8 pixels. Each image contains one artery lumen, and there are eight images in a sequence. The noise intensity is randomly given at every pixel, and the SNR (signal-to-noise ratio) is measured as such:

(6) $$\text{SNR}=\frac{1}{M\times M}\sum_{x=1}^{M}\sum_{y=1}^{M}20\log_{10}\frac{g(x,y)}{\left|n(x,y)\right|}$$

where M = 41 is the image size and g(*x,y*) and n(*x,y*) represent the raw image gray-level and added noise (zero mean, uniform distributed) intensity at coordinate (*x,y*), respectively, where |*n*(*x,y*)| ≤ *n_level_*. The SNR is controlled by *n_level_*, where 0 < *n_level_* < 1. The images are smoothed by a Gaussian function having σ = 0.5 before noises are added, which makes images more realistic.

To extract boundary information, we apply the directional gradient [6]. We investigate the optimal parameter, which is set to achieve the best accuracy of the proposed method for round shape boundary detection. There are two parameters to be explored: the image resize and weighting factors *s* = *α*_1_ = *α*_2_ in Equations (2a) and (2b). We fix the constraints as *d*_1_ = 1 and *d*_2_ = 2. The factor *d*_2_ is larger because we allow the method to catch larger radius changes in the sequence. The image resize is performed after the image gradient computation, and it is transformed from Cartesian to polar coordinates. A 3D volume containing the gradient images is then used as input for the 3D-expansion of DP.

### 3.2. MRA Real Images

A mobile MRI (Siemens-type “AvantoTM”, 1.5 T) was used for image acquisition [9,10]. Twelve of the forty-four participants in the TEFR09 study participated in this study after approval from the local ethics committee of Ulm University in accordance with the Declaration of Helsinki. Complete MRI sequences were acquired from all subjects. To validate the novel SFA (superficial femoral artery) lumen detection algorithm presented in this study, several MRI sequences from selected subjects were randomly selected.

**Figure 3.**
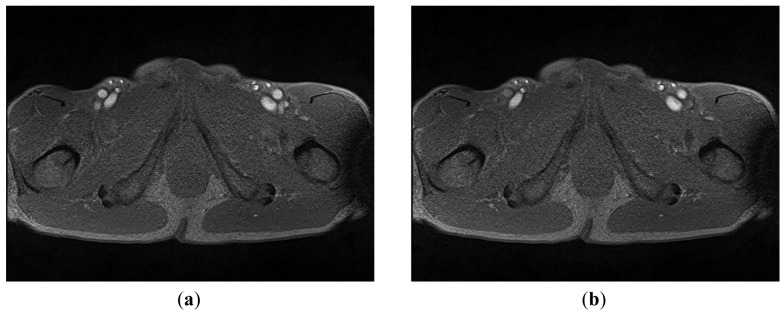
Two raw MRA images in one sequence. Each sequence contains 50 images. (**a**) Image taken in the systolic phase; (**b**) Image taken in the diastolic phase. The arrow indicates where the SFA is.

A mobile 1.5-T Magnetom (Siemens-Avanto™, Model Mob. MRI 02.05, Siemens Ltd., Erlangen, Germany), with a flexible 6-channel body matrix coil with 6 integrated low-noise preamplifiers (Siemens Ltd.) and bilateral table fixation, was used to acquire SFA MRI sequences from the subjects. All of the participants were athletes. The athletes were fixed in a stretched supine position, head forward on the MR table. To identify the axial perpendicular acquisition location at the right SFA 10 mm beneath the bifurcation of the common femoral artery, a biplane coronal and sagittal localizer (TRUFI: “true fast imaging with steady state precision”; Siemens Ltd.) was used. As in the common carotid artery MR-measurement, changes in the SFA diameter during systole and diastole was assessed with a T2*-weighted gradient-spoiled gradient-echo cine-sequence (FLASH: “fast low angle shot”, Siemens Ltd.) in a two-dimensional cross section view with prospective two-dimensional ECG gating (cardiac triggering). Specific sequence parameters were set thus: flip angle 15°, echo time variable between 4–6 ms (depending on heart rate), repetition time variable 20–40 ms (depending on heart rate), slice thickness 6 mm, field of view 768 cm^2^, matrix size 512 × 384, pixel width 0.625 mm (pixel area 0.3906 mm^2^) ISO, pixel bandwidth 250, and 50 images per sequence for one RR-cycle (approximately 300 heart beats per sequence). The total imaging acquisition time was approximately 4 min 30 s to 5 min for each sequence. Figure 3 shows two typical MRA images: 3(a) was taken in the systolic phase and 3(b) in the diastolic phase.

Each real MRA image sequence contains 50 images. Our previous study has proposed an automatic SFA position detection method [10]. An ROI (region of interest) can be extracted using that technique, in which the right SFA is in the center. The directional gradient [9] is then applied to the ROI to extract the boundary information. The extracted gradients are transformed from Cartesian to polar coordinates. Fifty total gradient images form a 3D volume to be input for the 3D DP.

### 3.3. Accuracy Analysis

The proposed system is applied, and the SFA cross-sectional lumen area of each image is calculated for the following comparison. The comparison is performed by calculating their relative unsigned errors:

(8) $$\varepsilon_{i}=\left|A_{\text{Manual}}\left(i\right)-A_{\text{Automated}}\left(i\right)\right|/A_{\text{Manual}}\left(i\right)\times 100\%$$

where *A_Automated_*(*i*) and *A_Manual_*(*i*) are areas calculated by the automated and manual drawing on image number *i*, respectively. The mean errors (on 50 images) and standard deviations (SD) can be calculated.

## 4. Results

**Figure 4.**
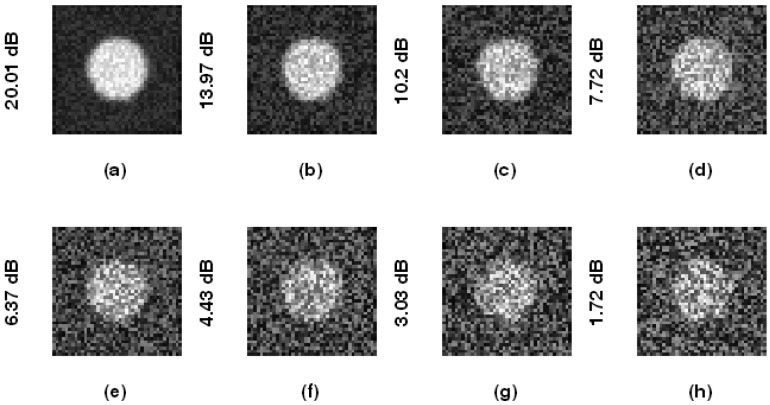
The phantom images with added noise from different SNRs.

**Figure 5.**
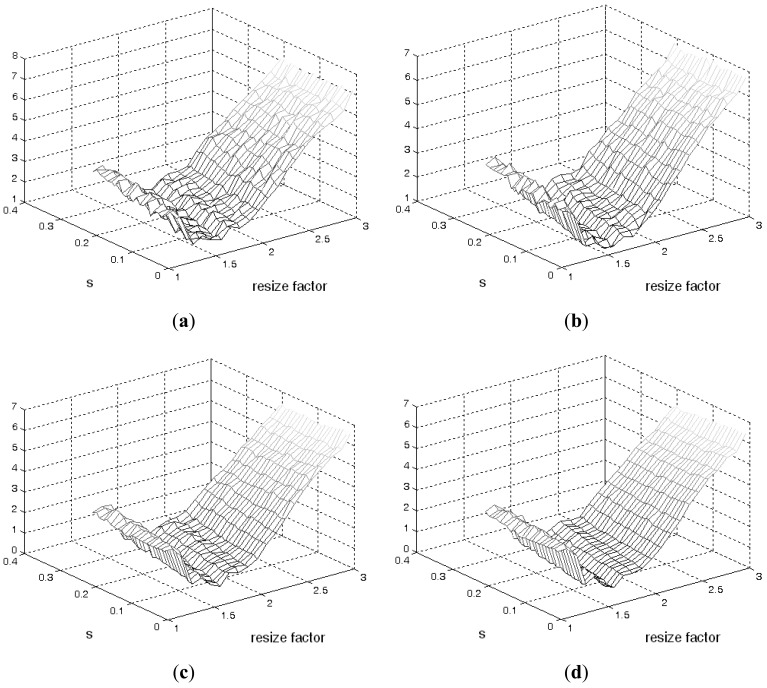
The unsigned error plots of different phantom images. SNR is (**a**) 14 dB, (**b**) 16 dB, (**c**) 18 dB, and (**d**) 20 dB.

**Figure 6.**
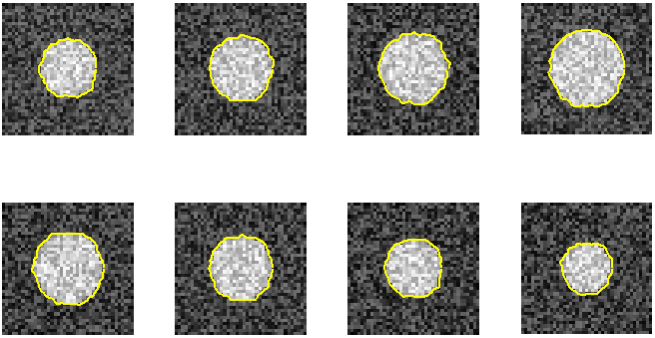
The contour detection results. SNR = 14 dB, s = 0.01, resize factor = 1.6. Averaged unsigned error = 1.5%.

Figure 4 illustrates phantom images with different noise levels (SNR). To investigate the effects that these two parameters (resize factor and weighting factor s) have on accuracy, four different phantom image sequences, with SNR = 14, 16, 18, and 20 dB, are tested. Figure 5 shows the corresponding averaged accuracies. The z-axis in Figure 5 denotes the averaged unsigned error percentage (for eight images and ten experimental repetitions). The value of s is discretized from 0.01 to 0.21 with a step of 0.01. The resize factor value is discretized from 1 to 3 with a step of 0.1. From the results, we found that parameter s does not affect accuracy too much but the image-resize factor does impact accuracy. The optimal image resize factor value is 1.6, no matter the noise level. When SNR = 14 dB, the unsigned error ranges from 1.50% to 2.47% with resize factor = 1.6. The best accuracy appears when s = 0.01. Without image resize (factor = 1), the unsigned error ranges from 3.15% to 4.21%. The best accuracy appears when s = 0.02. When SNR = 20 dB, the unsigned error ranges from 0.77% to 1.62% with resize factor = 1.6. Without image resize (factor = 1), the unsigned error ranges from 3.05% to 3.46%. The best accuracy appears when s = 0.06. Figure 6 demonstrates the contour detection results (SNR = 14 dB).

**Figure 7.**
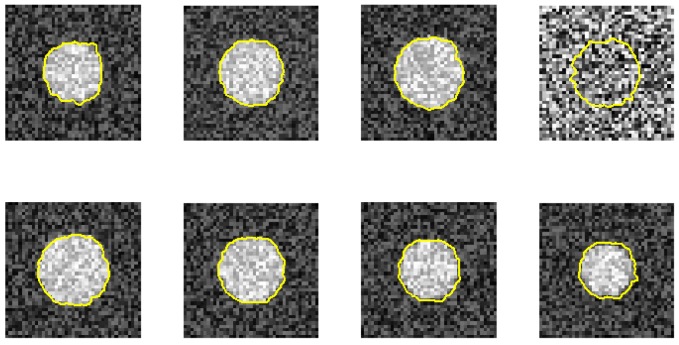
One image is ruined. The proposed method can still hold the contour because of the continuity of the 3D DP method (*d*_2_ = 1).

**Figure 8.**
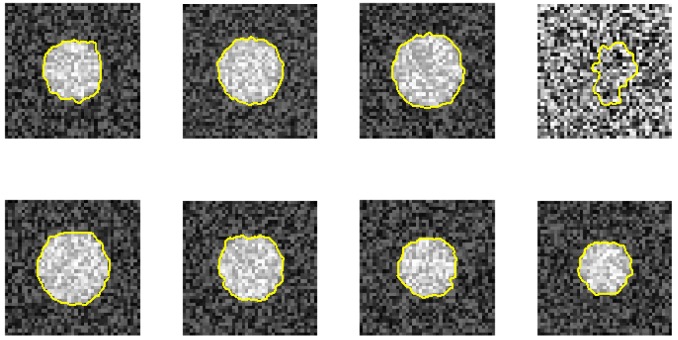
The traditional DP fails to hold the contour, as there is no information between image slices.

Furthermore, we demonstrate the ability of the proposed method when one image is ruined. Figures 7 and 8 use the same eight phantom images (SNR = 14 dB), and their boundary detection results differ on the ruined image. The traditional DP has no knowledge about the third direction, and it fails to detect the boundary if the image is ruined. However, our method shows its robustness against strong noise.

**Figure 9.**
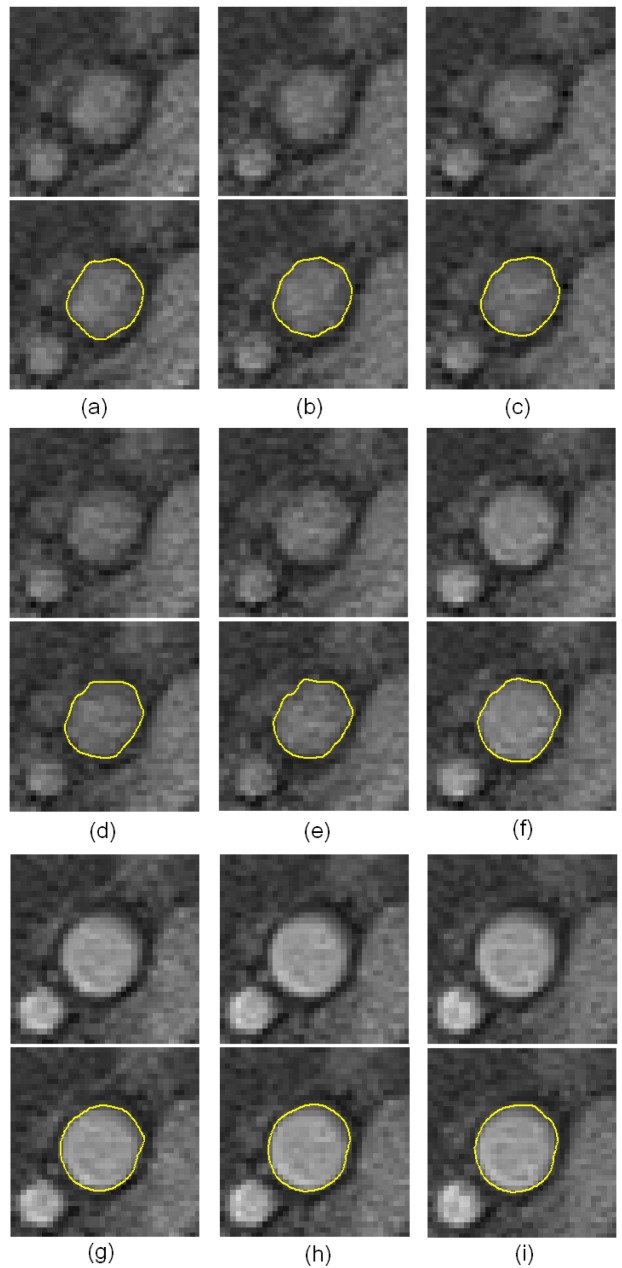
Boundary detection results. Nine sequential resultant images are shown. The vessel boundaries are vague in the first five images.Their exact boundaries are difficult to determine. Based on the continuity consideration in three dimensions, the proposed method can detect the correct vessel boundaries.

In the real MRA image study, we show the robustness of the proposed method against vagueness. Figure 9 shows the results of nine sequential images and their corresponding detected boundaries. They are organized in a 3-by-3 matrix. In each element of the matrix, the upper image is the raw sub-image, and the lower image has its result superimposed on it. From the results, we can see that the artery's boundaries are vague in the first five images. It is even difficult for a medical expert to define their boundaries. Our algorithm can overcome this difficulty by using the 3D dynamic programming that has considered the connectivity information in three dimensions in these vague images. The proposed method thus has superior performance against vagueness on the boundary location.

**Figure 10.**
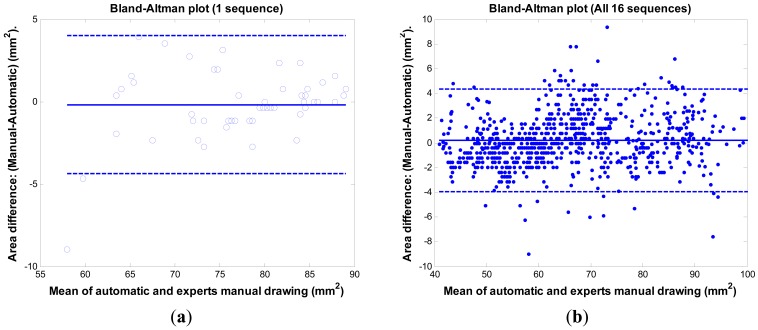
Bland-Altman plots. The solid line indicates the mean, and dash lines are ± 1.96 SD. (**a**) The plot of sequence no. 9 (50 images). (1.96 SD = 4.2 mm^2^); (**b**) The plot of all 16 sequences (800 images). (1.96 SD = 4.1 mm^2^).

The Bland-Altman plot [24] is often used in a clinical comparison of a new measurement technique with an established one to see whether they agree sufficiently for the new technique to replace the old. Figure 10 shows the comparison results between the proposed method and the experts' manual tracings via the Bland-Altman plot. Figure 10(a) is the result of one sequence having the mean error rate 2.1% ± 2.6%. The mean of the difference (y-axis) is −0.19 mm^2^ with SD 2.14 mm^2^. The 95% confidence intervals are within 4.2 mm^2^. Figure 10(b) is the result of 16 sequences (800 images) having the mean error rate 2.4% ± 2.0%. The mean of the difference (y-axis) is 0.22 mm^2^ with SD 2.11 mm^2^. The 95% confidence intervals are within 4.1 mm^2^. The bias is very limited (0.22 mm^2^). The system is consistent in its data number (SD = 2.11 mm^2^). The results demonstrate the system's ability to replace the expert's manual work.

**Figure 11.**
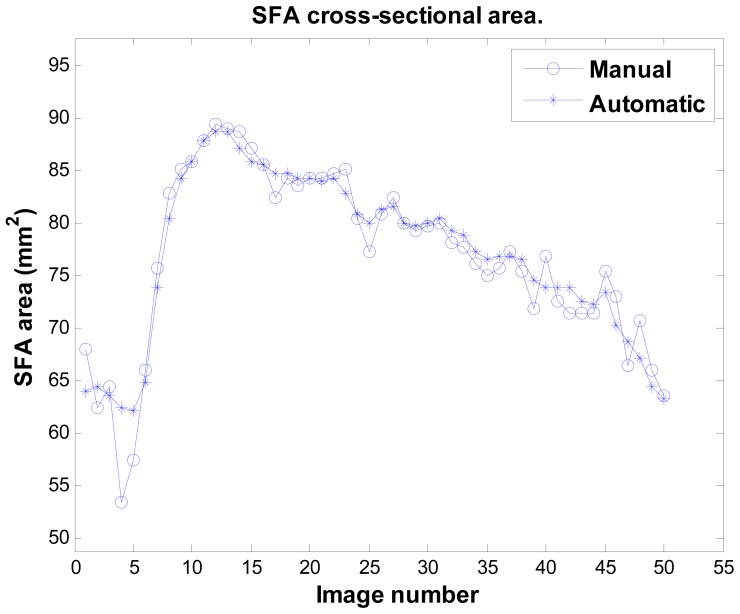
The cross-sectional SFA area changing in time (sequence no. 9). The unit in the y-axis is mm^2^. The x-axis unit is image number.

Figure 11 illustrates the result of sequence no. 9. Notably, the system can detect the artery's area decreasing (around image number 5 in this sequence), which is a physiological phenomenon. This is the most difficult issue, as the artery's boundary in this phase is unclear and it has the least image contrast. Sometimes even experts cannot exactly locate the boundary. Table 1 provides the accuracy analysis of the system using the unsigned errors rate of all 16 sequences. The maximum mean error rate is under 3.6%, and all SD are under 2.8%.

The computer system has an Intel^®^ Core™ 2 CPU T5600, 1.83 GHz, with 2 GB RAM. All programs are designed based on the Matlab platform [25]. The computation time for each sequence (50 images) is approximately 2.1 s. The computation is much faster than our previous study [10], in which it takes approximately 25 s under the same hardware construction.

## 5. Discussions

In the proposed algorithm, there are two weighting factors, *α*_1_ and *α*_2_, and two constraint factors, *d*_1_ and *d*_2_ (Equation (5)). The constraint factors allow the maximum neighboring boundary, whereas the weighting factors affect boundary continuity. Distance *d*_1_ defines the boundary connectivity on the same image. It is thus usually defined to be less than or equal to 2. Distance *d*_2_ defines the boundary connectivity on a different image level and is thus case dependent. If boundaries on different levels have a large distance, then *d*_2_ must be defined larger. In this study, the SFA have 50 images during an R-R interval. The boundary thus does not change much, and *d*_2_ is set to 2.

From the phantom study, we know that the effect of the resize factor is larger than the weighting factors *α*_1_ and *α*_2_. We therefore set the resize factor to 1.6 (the optimal parameter value in the phantom study) and choose the weighting factors as 0.1 (*α*_1_ = *α*_2_ = s = 0.1) arbitrarily in the real MRI study.

We have proposed a three dimensional DP, which can find an optimal surface in a 3D matrix where features are normally saved. This method has robustness against strong noise, especially when one or two images are absent in the image sequence. The proposed property is useful, especially for detecting an artery's boundary in an MRI when one or two images in the artery's boundary are vague. The connectivity can help rebuild the artery's boundary. This method can be extended to track an object's boundary in image sequences, especially when partial occlusion exists. Furthermore, it is possible to embed another model into the DP and let it extract a special shape, including a round [6] or oval shape. The limitation of this proposed system is that it cannot handle branches. Many vessel segmentation methods must face the branching problem; fortunately, we do not have that problem in this study.

**Figure 12.**
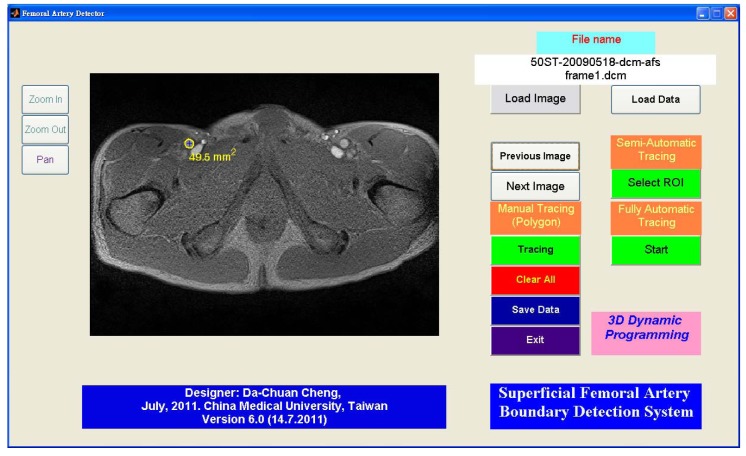
The software system GUI. The system can read DICOM and other image formats supported by MatLab. However, only the DICOM format offers pixel size information.

Figure 12 shows the GUI (graphic user interface) of this system. The semi-automated and fully automated algorithms and the manual tracing function are embedded together. The GUI has zoom functions; it thus allows the possibility of manual tracing the sub-pixel resolution to trace the boundary. The semi-automated algorithm lets the user select an arbitrary vessel (using ROI selection: region of interest) for boundary detection. The software is designed for detecting objects with a brighter area than its surroundings. Detecting the dark area is possible by inverting the gradient images.

The applications of the proposed algorithm are not restricted to vessel segmentation. It is also practical for bone segmentation in 3D CT slices [26] and detecting the boundaries of intima and adventitia of common carotid artery inner walls in dynamic sonographic image sequences [6], which requires high segmentation accuracy. The advantage is that this method provides the connectivity information in three dimensions. Many segmentation methods do not consider the third dimension. The computation is also fast in this method, so real-time implementation on the object tracking problem might be possible.

Our future aim is to test this system on sonographic image sequences to detect the intima on both sides of the CCA inner wall to extract the inner wall lumen diameter changing with time. Combined with the blood pressure information, the lumen diameter change can compute the arterial elasticity, which is an important marker in clinics. As a final remark, we must note that this method might be extended to the dual surface detection in a 3D volume, such as the dual dynamic programming in a 2D case [8].

## 6. Conclusions

We have developed a novel algorithm that can detect the optimal surface in a 3D volume matrix. This algorithm is applied to detect SFA boundaries in MRA image sequences. According to our phantom study, we conclude that the optimal parameter values for using the 3D DP are thus: image resize factor = 1.6, and weighting factor s = 0.02. In the MRA real image study, the average relative unsigned error is 2.4% ± 2.0% in 16 MRI sequences (800 images) compared to the manual tracings. The algorithm proposed in this study is reliable with repeatable results that can replace the experts' manual work.

## Figures and Tables

**Table 1.** The average unsigned relative errors on 16 MRI sequences (800 total images).

**Sequence No.**	**Mean (%)**	**SD (%)**	**Sequence No.**	**Mean (%)**	**SD (%)**
1	2.4	1.8	9	2.1	2.6
2	3.2	2.1	10	2.9	2.5
3	1.4	1.3	11	1.4	1.0
4	3.2	2.7	12	2.5	1.7
5	3.6	2.6	13	2.4	1.2
6	3.1	2.4	14	3.2	2.0
7	1.9	1.6	15	1.6	1.7
8	2.7	2.8	16	1.4	1.1
**Mean**	**2.4**		**SD**	**2.0**	

Each sequence contains 50 images. Resize factor = 1.6, s = 0.1.
